# The benefits for health care staff of involvement in applied health research: a scoping review

**DOI:** 10.1186/s12961-025-01365-1

**Published:** 2025-08-18

**Authors:** Andria Hanbury, Emily Parker, Rebecca Lawton, Jayne Marran, Jane Schofield, Laurie Cave, Lynn McVey, Emma Eyers, Peter Van der Graaf, Roman Kislov

**Affiliations:** 1 Bradford Institute for Health Research, Bradford, England; 2 University of Leeds and Department of Health and Social Care, London, UK; 3 University of Leeds and Bradford Institute for Health Research, Bradford, England; 4 Bradford Teaching Hospital NHS Foundation Trust, Bradford, England; 5 University of Leeds, Leeds, England; 6 Northumbria University, Newcastle, England; 7 Manchester Metropolitan University, Manchester, England

**Keywords:** Health care staff, Research involvement, Benefits

## Abstract

**Background:**

Initiatives are increasingly encouraging health and social care staff involvement in research, with evidence for patient and organisational level benefits. There is less evidence of the benefits for staff and whether this varies by type of involvement. This scoping review aimed to identify the different ways staff are involved in applied health research, the benefits experienced, and whether this varies by type of involvement. This will help to inform leaders in service organisations, funders, and researchers about how to maximise such benefits.

**Methods:**

The scoping review followed the JBI methodology. Four databases were searched: CINAHL, MEDLINE, PsycINFO and Scopus. Grey literature was identified via Google, Google Scholar and relevant websites. Records had to be UK-based, published in English between 2003 and 2023 and cover applied health and care research, health care staff involvement and report on benefits. Text was extracted from records, coded afterwards, and quality checked. The benefits were distilled by four research active health care staff. Descriptive statistics and narrative synthesis were used to report the results.

**Findings:**

In total, 49 records were reviewed, 42 records were from the database search and 7 from the grey literature search. Records were most commonly journal articles (*n* = 44), covering multiple care settings (*n* = 15) and mixed professional groups (*n* = 24), used qualitative methods (*n* = 22) and focussed on clinical academic roles (*n* = 21). Six benefits of involvement in research were distilled: personal fulfilment, general competencies/skills, connections/networks, opportunities for learning, opportunities for leading improvements in practice, and using evidence more effectively. Records that focussed on the more intensive clinical academic roles reported more examples of opportunities for leading improvements in practice, and the building of connections and social support. Non-clinical academic records more frequently reported that involvement in research provided opportunities for learning.

**Conclusions:**

These findings support efforts to involve staff in research, with a range of benefits associated with enhanced job satisfaction, even when research involvement is in a less intense form, such as participation in a study. These findings can be used to encourage involvement, with recommendations for future research to review the benefits for social care staff, and to examine more directly the effect on staff wellbeing and retention.

**Supplementary Information:**

The online version contains supplementary material available at 10.1186/s12961-025-01365-1.

## Introduction

Positive associations are reported between clinicians’ and health care organisations’ engagement in research and improved processes of care (for example, treatment access, adherence to best practice) and patient health outcomes, with a potential ‘dose effect’ of research engagement also suggested [4, 5]. Similar positive associations have been reported for research active English general practices, including their scoring higher on measures of clinical quality of care and reduced accident and emergency attendances [13]. Many of these advantages are realised through the positive influence research involvement can exercise on translation of research evidence into day-to-day care, as well as the organisation and delivery of services more broadly [54], as well as through the value of research networks which have a remit to reach out to and engage clinicians [5]. Perhaps unsurprisingly then, there is a growing onus on supporting health and social care staff involvement in research. For example, in England, the National Institute for Health and Care Research (NIHR) announced (2023) an additional £30 million a year of funding to expand and strengthen existing opportunities for health and social care professionals to develop research careers. Examples include fellowship schemes at pre- and post-doctoral levels, and the NIHR INSIGHT programme, providing funding to inspire students into research. In the UK, as well as the USA and Australia there are also advanced clinical practice roles, with research in the job specification, and research is specified in competency frameworks of the Nursing and Midwifery Council (NMC, n.d.).

Underpinned by a rich and expanding body of literature on co-production, including works from the social sciences and humanities [14], political science [6], public management [12], and academic entrepreneurship [38], scholars have been equally enthusiastic in creating a variety of conceptual frameworks, guidelines, and principles for co-production. Recent systematic reviews of co-production have summarised the different co-production approaches in use and collated the outcomes and effects of co-production (Slatterly et al., 2020), [47]. Despite the proliferation of conceptual thought, empirical studies on co-production are less frequent [42]. Many co-production models and frameworks are not supported by robust evidence [53] and do not describe in practical terms what co-production of research on the ground looks like [47].

Similarly, whilst the benefits of research involvement for organisations and patients are well reported, less is known about the benefits for staff themselves, although the challenges to involvement are better reported (for example, [23, 24, 41, 49]). Where benefits are reported, as found by Marjanovic et al. [29], the tendency is towards commentary on the potential benefits, rather than actual benefits identified for example, through interviews or surveys with staff. Importantly, there may also be ‘hidden benefits’ for health and social care staff. Hidden benefits for patients involved in research include their having improved knowledge of their health condition, a sense of pride from taking part, an increased desire to help others, and appreciating the opportunity to talk to someone [7, 16, 50].

For health professionals, hidden benefits may be linked to the process of being involved, such as satisfaction and a sense of achievement. There is potential for such hidden benefits for staff to contribute to job satisfaction and hence improved workforce retention. Indeed, in the NHS Long Term Workforce plan [36], the word ‘research’ appears 29 times, including reference to enabling more flexible and autonomous career development through portfolio careers encompassing research roles for medics. Workforce retention is a pressing issue, with the current NHS vacancy rate of 6.9% (March 2024, NHS England, [35]) across all roles, which is even higher for nurses and midwives (7.5%). Thus, given this backdrop of stretched resources, it is vital to be able to demonstrate the full plethora of benefits if staff are to be supported to spend time on activities other than direct patient care. It is also vital to help identify novel approaches to upskill, retain and attract new staff.

This scoping review was undertaken to identify the different types of involvement in research, what the benefits are, including hidden benefits, and whether they vary depending on the type/intensity of involvement. We specifically explored the benefits because barriers to involvement in research are already well reported, and we focused on involvement in applied health and social care research, rather than on clinical research, as this is less well explored. The findings will be of value to researchers seeking to encourage and optimise health care staff involvement, to the NHS, and to funders and policy makers seeking to attract health care staff into research and to understand and maximize the benefits for them.

## Method

The JBI methodology for scoping reviews was followed [39], beginning with a protocol, with subsequent reporting guided by the Preferred Reporting Items for Systematic Reviews and Meta-Analyses (PRISMA) Extension for Scoping Reviews (PRISMA-ScR).

The review questions were:

1. What are the different ways that health care staff can be involved in applied health and social care research?
2. What are the benefits to health care staff of being involved in applied health and social care research?
3. Do health care staff experience different benefits of research involvement depending on the type/level of involvement?

### Identifying relevant records: search strategy and screening process

The search strategy was limited to the UK. This UK restriction was due to the review team comprising researchers and leaders in improvement and implementation science from five National Institute for Health Research (NIHR) funded Applied Research Collaborations. The aim was to gain insights for wider learning across the NIHR infrastructure, as well as beyond to the UK NHS. The search strategy covered database-published and grey literature-identified sources. For the database search, a preliminary search of MEDLINE was undertaken in January 2023: the text words in the titles and abstracts and the index terms used to describe the articles informed development of the final search strategy for MEDLINE (see Additional file 1). This was then adapted for the following databases: CINAHL, PsycINFO and Scopus. For the grey literature search (Additional file 1), the following sources were searched:

1. Google and google scholar, with the restriction to only review the first 100 records
2. Websites of:
  - National Institute for Health Research
  - Council for Allied Health Professions Research
  - UK Research and Innovation
  - Council of Deans of Health Clinical Academic Roles Implementation Network
  - Department of Health and Social Care
  - Health Education England
  - The Kings Fund
  - The Health Care Improvement Studies Institute
  - Florence Nightingale Foundation

Collaborators with expertise in NHS research capacity building (the Addressing Capacity in Organisations to do Research Network in Yorkshire and Humber), a patient panel, and the wider research team reviewed the search strategy.

The searches were run in each database. All search returns (records) were transferred into Covidence, an online screening and extraction tool [11]. Duplicates were removed. Using the inclusion/exclusion criteria in Table 1 below, title and abstract screening was undertaken by two researchers (EP, LC) who double screened all records at this stage. Cohen’s Kappa was calculated to check consistency, with disagreements resolved through discussion. Full text screening was undertaken by three researchers (EP, JS and JM). Initially, a 10% sample was screened by all three, with Cohen’s Kappa checked and any disagreements resolved through discussion. Following this, the remaining references were split between the three researchers. The reason for exclusion was noted, in the order: context (UK, followed by applied health research), population, outcomes (benefits), and record type. For any records where there was uncertainty, the three researchers met to discuss the decision. The grey literature search was then run, with single screening split between three researchers (AH, JS and JM). Any records where there was uncertainty were flagged and reviewed by one of the other researchers.

**Table 1:** Inclusion and exclusion criteria

Criteria	Inclusion	Exclusion
Population	Allied Health Professional (AHP) roles defined by NHS England [34] or other registered staff roles within the UK NHS: doctors, nurses, midwives, social workers, health scientists, clinical psychologists, dentists, dental nurses and practitioners, pharmacists and pharmacy assistants, therapists, podiatrists, dietitians, occupational therapists, operating department practitioners, orthoptists, osteopaths, paramedics, physiotherapists, prosthetists and orthotists, radiographers and speech and language therapists	Non-registered health and social care staff, including domestic staff, porters, healthcare assistants and administrative teams
Context	Applied health and/or social care research (for example, health services research not involving drug trials or biomedical studies) UK AND all types of involvement (for example, as a member of a co-design team, as a co-applicant, project advisor, project lead, PI in an organisation, research secondment, internship, fellowship, PhD, mentor, research champion, research participant, etc.)	Involvement in clinical research only (defined as drug trials or biomedical studies) Non UK records
Outcomes	Benefits reported either directly (such as via survey or qualitative interviews) or via an author/s reflecting on their experiences or using illustrative case studies, even if benefits was not main focus of record	Benefits only discussed in relation to staff using research (for example, engaging in evidence-based practice) or positive outcomes linked to a research intervention rather than the research process itself Motivators for research involvement only, or conjecture regarding benefits rather than directly reported or reflected upon
Sources	Journal articles, including opinion pieces and editorials, and grey literature including blogs, conference posters and reports (provided cover benefits rather than purely strategy focussed)	Reviews
Restrictions	English language only Date range 2003–2023*	Not in English language Pre 2003

### Eligibility criteria

Table 1 summarises the inclusion and exclusion criteria. This was applied to both the database and grey literature search identified results.

### Data charting: data extraction

For the retained records only, the variables summarised in Table 2 were extracted into an excel table:

**Table 2:** Extracted variables

Variable	Details/coding used
Record details	Authors, title, year, reviewer’s name
Type of record	Journal article, blog, commentary, report, poster
Research design, where applicable	Qualitative, quantitative, mixed methods, other
Population	Doctors, nurses, midwives, allied health professionals, social care staff, pharmacists, mixed, unclear
Context of care/setting	Community care, mental health, obstetrics/gynaecology, oncology, paediatrics, podiatry, primary care, respiratory, rheumatology, speech, and language, mixed, unclear or not reported or not specific
Type of involvement	Clinical academic (defined as per NHS England as a dual role; a clinical professional combining their clinical role with a research career in academia. Where the term ‘clinical academic’ was used, this was counted as a clinical academic without interrogating the role description); mentoring/internship/scholarship activities; research recruitment, delivery, or data collection or interpretation tasks; research as part of role (intervention development and including research champion roles but excluding formal clinical academic roles); research participant; research training (below the level of a scholarship) and ‘unclear’
Organisations supporting/coordinating	Whether a centrally funded national programme (for example, Health Education England, or National Institute for Health Research) or not (for example, NHS internally funded, or where a small pot of funding has been applied for)
Reported benefits	Free text

To ensure transparency, the variables of research design, population, region, and context, type of involvement, organisations and benefits were initially extracted as free text. The free text was reviewed, and coding categories then developed to ensure good coverage. The coded variables were entered as new columns, alongside the free text data. The review and coding was undertaken by two researchers (LM and AH). A third reviewer (EE) did a quality check of 10 records’ coding (20%), comparing the free text with the coded summaries, with any disagreements resolved by discussion. Extraction and coding was also discussed at a wider research group monthly meeting.

### Synthesis of data

To address review questions one and two, for each variable, the distilled codes were summarised across all records. For the benefits, a 1.5-h online group consensus session was run, involving four health care staff with experience of applied health research and part of the wider research team. This was to ensure our coding for these richer extracts (compared with extracts regarding type of involvement and professional group, for instance) was sense checked by people with research and clinical experience, reducing the risk of nuances in benefits, for example, being ignored. Extracts from each record, summarising the benefits, were shared with the group. Individually, participants were requested to read the extracts and divide them into thematic categories before arriving at consensus about the categorisations and agreeing on headings for the final categories.

To address review question three, pivot tables were created in excel by AH, summarising coded benefits by coded type/level of involvement. Owing to the number of benefits distilled from the free text data and types of involvement, some combining of coding categories was necessary for these comparisons due to the small numbers in certain categories. Therefore, the most intensive level of involvement-clinical academic roles-was compared with all other types or levels of involvement combined. The pivot tables were generated to highlight differences and similarities across groups, enriched through reference to the accompanying free text columns, rather than for formal statistical comparison given the small numbers.

## Results

**Fig. 1:**
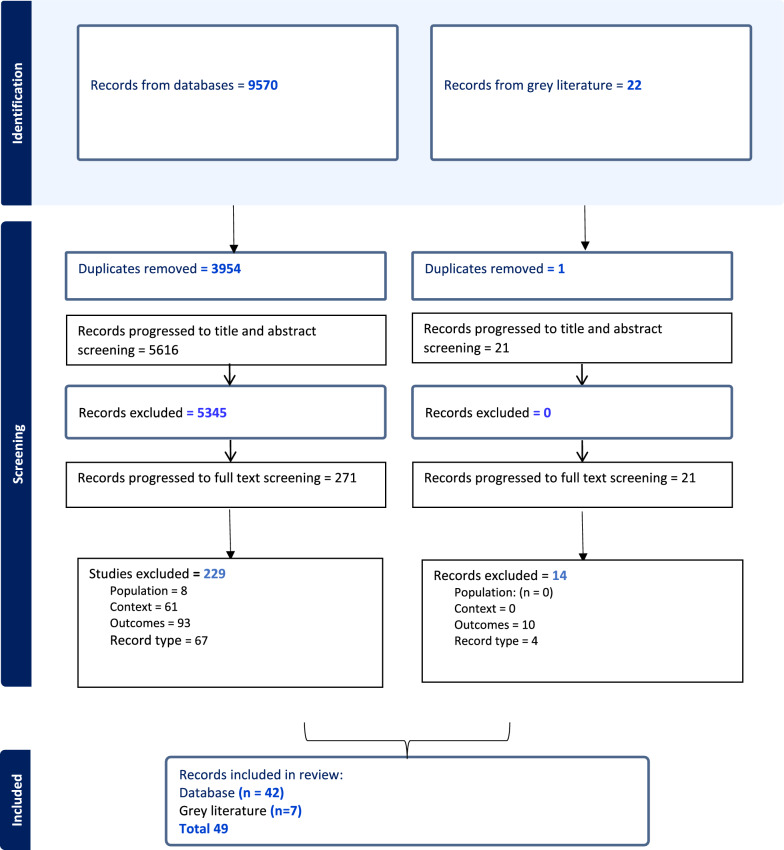
PRISMA diagram

In this section, we begin with reporting on characteristics of the retained records, before covering each research question. The number of records retained for the database and grey literature components of the review are summarised in a PRISMA diagram (Fig. 1).

### Characteristics of records

49 records were retained across the database (*n* = 42) and grey literature (*n* = 7) identified records. The title and abstract screening Kappa was 0.52, and the full text screening Kappa was 0.69. Of the retained records, there were 44 journal articles, 2 blogs, 2 reports and 1 poster. These covered direct reports of benefits (*n* = 38), such as via survey results or insights form qualitative interviews, versus more reflective (*n* = 11), for example, an author reflecting on the benefits they have gained. A range of clinical care settings were covered, including mental health care (*n* = 2), community care (*n* = 2) and paediatrics (*n* = 4). The majority of records, however, detailed research or activities involving multiple care settings (*n* = 14).

21 of the 49 records used qualitative methods to explore the benefits for staff, followed by mixed methods (*n* = 12). Only five records used quantitative study designs on their own. The remaining 11 were reflective papers, commenting on experiences or case study examples. The most common professional grouping was ‘mixed’ (*n* = 24), of which *n* = 20 included nurses mixed with other professionals. Nurses were also the most commonly represented single professional group (*n* = 10), followed by allied health professionals (*n* = 5). Doctors were represented in four studies where they were the only professional group involved, followed by midwives (*n* = 3), and community pharmacists (*n* = 2). In one record, it was unclear (‘clinicians’).

For the 31 records where funding for the research involvement was mentioned/clear, 18 reported central, national funding as the source (for example, Health Education England and NIHR funded initiatives including internships), often in collaboration with the NHS and/or a higher education institution. The non-centrally funded initiatives included examples of NHS initiatives to develop more research orientated roles, internships and scholarships, higher education initiatives to develop internships for AHPs, and a charity funded scholarship.

### What are the different ways that health care staff can be involved in applied health and social care research?

Arguably, the most intense forms of involvement identified in the review were clinical academic roles (the largest proportion of records, *n* = 21), and research training, mentoring/internships, and scholarships (*n* = 10). Clinical academic examples include mixed research and teaching, research-only, and research and clinical roles (for example, [52]), and a clinical academic ‘bridging scheme’, funded by Health Education England to support clinicians develop the necessary skills for a clinical academic role (for example, [19]). Examples of research training and mentoring type of roles include a hospital trust organised clinical improvement scholarship programme lasting a year which involved clinicians conducting their own research [2] and a mentoring scheme for research training [55]. Other types of moderately intensive involvement were where research was described as a substantial part of the role but it was not a formal clinical academic role (*N* = 6), for example, a research champion role which was designed to reduce barriers to research and promote research at a specialist mental health and community services trust [18], and nurse involvement in the delivery of a complex intervention [3]. Finally, examples of *relatively light-touch involvement* included participation in research recruitment, delivery, or data collection activities (*n* = 4), such as conducting peer interviews (for example, [31]) and supporting an intervention pilot study through recruiting participants [48], research participation (*n* = 5).

Additional file 2 summarises the retained records and key characteristics. **Please insert here.**

### What are the benefits to health care staff of being involved in applied health and social care research?

The benefits were distilled into six overarching themes.

1. Personal and professional fulfilment, including career development.
  This was the most frequently reported benefit, cited in 34/49 of the records. Being involved in research was reported in six of the records as leading people into further study (for example, fellowships, internships, and PhDs), or giving them the desire to do so (six records). It was also linked to career progression (seven records) or anticipated future career impact (six records). This was suggested to be through the research skills developed, but also through increased confidence cited in several records (for example, [17, 57]) and through increased opportunities and insights into careers involving research, for example, the potential to combine practice, management, research, and teaching (for example, [43]). The latter covered a change of direction towards clinical academic or clinical research roles or being able to ‘carve’ one’s own career path out (for example, [21]). Records also provided examples of people applying for, and gaining, research funding, co-authoring publications, and conference presentations. Some records discussed examples of research involvement being transformative and life changing for people, and of the job satisfaction, enjoyment and excitement afforded by research. This was linked to morale and engagement and in one instance reportedly resulted in a person retracting their resignation (see [56]). There were mentions of intellectual stimulation, of people thriving, and of personal achievement and pride. Complementing this, some records suggested research involvement to act as a good buffer to the stresses of full clinical practice, enabling the best of both worlds, or (in the case of participating in a research interview) to act in a cathartic way, giving people a chance to reflect on their practice during the Covid-19 outbreak. Finally, two records (Code et al., 2019, and [51]) touched upon female empowerment, in terms of the enjoyment of seeing other women furthering midwifery knowledge through their research, of showcasing to their own children how women can have a science career and also commenting on the flexibility afforded by fellowships when raising a young family.
2. Opportunities for leading improvements in practice, at a local and national level.
  Overall, 32 of the records cited examples of this benefit. Healthcare staff typically made general comments and assertions regarding the link between research and better care or anticipated there to be a positive future impact of ongoing and/or recently completed pieces of work. However, some tangible examples were provided in eight records. For example, one record noted that research-active clinicians had helped develop national treatment guidelines [33], another highlighted how an intern had developed a strategy to improve end of life care at their trust which was being implemented by a working group [20], and another provided an example of small but impactful changes to help improve the experience of end-of-life care for relatives [9]. There were three records that discussed examples of improved patient-centred care and experience through better communication skills [1, 3, 48]. Other records in this theme highlighted more generally how research involvement had led staff to question practice more (for example, diagnoses and treatment choices, for example, [57]) and identify areas where there was scope for improvements to be made (for example, [8]).
3. Building connections/networks, and social support.
  Twenty-four records cited examples of building connections and networks, but particularly the social support that came from this. Being involved in research was perceived to be associated with the potential to enthuse and influence others, with 11 records highlighting this—for example, demonstrating the benefits to colleagues on the ‘front line’ and supporting other’s development (for example, [9, 52]). This included not only their immediate team but also interns and students motivated to consider research through their provision of mentoring and support (for example, [8, 40]), as well as new staff members. There were also examples of involvement in research groups and special interest groups which appeared highly valued, and reference to general networking, including at conferences. These were described as supportive opportunities, reduced the risk of isolation, were inspiring, and provided an opportunity to learn more. References were made to the benefits of mixing with academics or previous interns and clinical academics, through a supervisory or mentor relationship, with support gained in how to balance competing demands of dual roles (for example, [19, 32]). This suggests that mixing clinical and research responsibilities might be more achievable with social support from others (in similar positions). Within all of this, there was reference to widened networks, for example, with senior staff (for example, [45]) and staff from different clinical professional groups (breaking down typical boundaries, for example, and people beyond their own organisation/UK wide, due to their shared interests. The emphasis was on the benefits of mixing with like-minded and inspiring individuals and widening networks. The records that focussed on clinical academic roles reported more examples of building connections and networks and social support (*n* = 14, 67%) compared with the other records (*n* = 9, 36%).
4. Opportunities for learning.
  Opportunities for learning covered two main facets: clinical practice insights and knowledge (10 records) and research skills and knowledge (13 records). Examples of how research involvement had benefited clinical knowledge include increased confidence to handle inappropriate requests for antibiotics [1] following participation in a workshop, a richer understanding of what it is like to live with malignant pleural effusion following co-production of a decision support tool [15], and insights gained by a clinical academic through visiting a range of teenage and young adult cancer services [26]. Healthcare staff also developed research skills including literature searching and critical appraisal, qualitative methods, quality improvement and evaluation skills (for example, [37, 55]). Examples of research knowledge included an appreciation and understanding of theory and its role in research, and an understanding of the place of research within health care policy. One record [10] noted a benefit of research training to be the ability to develop skills and knowledge in a safe space, enabling people to have exposure to research and the skills development, without having to wholly commit at that stage. Compared with the other benefits, there was more of a noticeable difference in the number of clinical academic focussed records versus non-clinical academic focussed records reporting this benefit, seven clinical academic focussed records (24%, cited this benefit, with two mentioning clinical practice and insights and four mentioning research skills and knowledge, and 1 record mentioning both types. For the remaining records, 15 (52% mentioned this benefit, split between eight citing research skills and seven clinical skills/knowledge examples.
5. Gaining general competencies and skills, beyond research.
  Being involved in research was perceived to support development of a wide range of general competencies and skills, many of which are applicable outside of research. Examples were cited in 18 records. Six records cited organisational skills, such as project and time management, gained through experience of, for example, managing one’s own workload and working more autonomously (three records), as well as through experience of training other staff (one record). Records also reported using more critical thinking and problem-solving skills (three records) and being more innovative and entrepreneurial [43], for clinical academics). Having a more self-driven or resilient approach (three records, for example, [28, 51], for example, being better able to respond to changing demands such as rapidly changing guidelines during the covid-19 pandemic was also mentioned. Examples of more interpersonal skills include enhanced communications skills (three records), including confidence communicating with colleagues and senior staff but also including increased confidence in openly discussing with patients and colleagues if there is uncertainty over management options [33] and leadership skills (four records) including change management.
6. Using evidence more effectively.
  Fourteen records highlighted how involvement in research had raised awareness of and enabled health care professionals to better understand the importance of evidence-based practice, and to feel confident in this area (for example, [2]. Records cited research helping staff to access and critically appraise evidence, and to use this to identify and address clinically relevant questions in clinical practice (for example, [8]). The latter included examples of seeking out evidence, questioning assumptions, as well as updating practice through literature review evidence. Two records also suggested evidence-based practice to be a ‘driver’ for some to engage in research, and three records touched upon influencing colleagues in this area, harnessing a culture of evidence-based practice, discussing evidence with colleagues [9, 19, 52].

### Do health care staff experience different benefits of research involvement depending on the type of involvement?

For each benefit, Table 3 below summarises the number and percentage of records focussed on the more intensive levels of involvement—clinical academic roles—compared with the less intensive, non-clinical academic roles that reported the benefit.

**Table 3:** Benefits by type/level of involvement

Benefit	% of more intensive (clinical academic) records reporting this (n records out of 21)	% of less intensive (not clinical academic) records reporting this (n records out of 25)
Personal and professional fulfilment	71 (15)	68 (17)
Leadership for improvements to practice at local and national level	71 (15)	60 (15)
Building connections and networks	67 (14)	36 (9)
Opportunities for learning	33 (7)	52 (13)
Gaining general competencies and skills	43 (9)	32 (8)
Using evidence more effectively	33 (7)	28 (7)

For certain benefits—personal and professional fulfilment, gaining general competencies and skills, and using evidence more effectively—the proportion of records reporting them was similar across those focused on clinical academic roles and those focused on other less intense levels of involvement. The more intensive clinical academic focused records reported research involvement more frequently to contribute to leadership improvements to practice (71% compared with 60%), and to have helped build connections and networks and social support (67% compared to 36%). Nonetheless, these patterns should be interpreted with caution, given the small numbers and lack of inferential testing. Records focused on the less intensive non-clinical academic types of involvement more frequently reported involvement in research to have provided opportunities for learning (52% compared with 33%). Again, this should be interpreted with caution, given the small numbers and lack of inferential testing. Taking the most frequently reported benefit, personal and professional fulfilment, and examining the arguably least intense form of involvement—research participation-3 of the records still reported this benefit. Examples include general practitioners who participated in a study and reported greater personal satisfaction from implementing skills they had learnt, alongside nurses who reported feeling more empowered [1], and reflections of health care staff who had participated in a research study, suggesting they had felt supported and engaged in the opportunity to have training about, and to discuss difficult situations (Sattar et al., 2022).

## Discussion

This scoping review aimed to identify the different ways health care staff are involved in research, the benefits of that involvement, and compared them across different types of involvement (from more to less intense involvement). Forty-nine records met the inclusion criteria. The 49 records, cover a mix of care settings and professional groups. Six different types of benefits were distilled, which include but go beyond career progression. They cover arguably more hidden benefits in the shape of personal and professional fulfilment, as well as creating opportunities for leading improvements in practice. Records provided examples of people thriving on research, the buffering effects of research compared with the stresses of daily clinical practice, and the development of communication, project management and leadership skills alongside research skills, such as literature searching. This, in turn, raised confidence in their ability to provide evidence-based practice.

When comparing benefits reported by records focussed on clinical academic roles—more intense forms of involvement—versus all other (considered less intense) forms—the frequency with which gaining general skills and competencies, personal and professional fulfilment, and using evidence effectively were reported was similar. However, records focussed on clinical academics reported fewer examples of opportunities for learning (the development of research skills but also clinical insights afforded by involvement in research), yet more examples of building connections/networks and social support, and more examples of opportunities for leading improvements in practice. The less frequent reporting of opportunities for learning was an unexpected finding. This may reflect the fact that clinical academic roles have work-loaded time for research and training and, as such, opportunities for learning may be a taken for granted benefit, compared with the opportunities for learning afforded by other types of involvement, such as recruitment and data collection. To some extent this difference may also reflect the range of different types of involvement in the non-clinical academic (less intense) grouping; from ‘just’ participation in research (still providing staff with a chance to reflect on practice and offering a cathartic opportunity), through to more involved scholarships and internships. Nonetheless, when the more granular examination of the records was made, focused on research participation only, there were still examples of personal and professional fulfilment from research involvement. This indicates that even the least intense forms of involvement were linked to benefits for staff, contributing to job satisfaction and personal satisfaction. Future research would benefit from developing a clear and more nuanced framework for assessing involvement according to type and intensity of activity, and stage of the research and knowledge mobilisation process.

The findings, thus, suggest that it is worthwhile to continue to create varied opportunities for research involvement, across all stages of the research process and from the more to the less intensive. Efforts should also be made to promote this broad range of benefits to encourage staff to become involved in research, beyond career development, building on the staff motivations uncovered by Marjanovic et al. [29] in their review. Indeed, some of the records reported health care staff as saying their involvement in research had been ‘life changing’ [44] and spoke of a sense of pride and achievement [32]. Given the ongoing challenge around workforce retention rates that include sickness, extended absence, and burnout (Garratt et al., 2024, the potential for improved satisfaction at work and the potential buffering effect of having some research time away from frontline clinical duties identified from this review has potential to contribute to improved staff retention. Indeed, actively supporting the wider health care workforce to engage with research, building connections and networks, and bringing outside interests into the workplace can help foster a feeling of membership for staff [25] which might help promote individual wellbeing and underpin local and national workforce retention policy. Providing a range of different involvement options will also suit different people and encourage more staff to consider research opportunities, for example, taking note of the records that highlighted how research was seen to provide greater flexibility for people with young children by one respondent, the satisfaction of seeing women contributing to midwifery care through research, and showing how women can have science-based careers. Thus, identifying, comparing, and evaluating a range of strategies deployed to promote involvement of staff in research would provide a fruitful avenue for future enquiry. This should include the level of influence and power afforded to staff, a more nuanced consideration beyond ‘type of involvement/role’, and how these opportunities can be spread equally across the workforce. This should reduce the risk of contributing to gender inequalities in healthcare staff career pathways and avoid further adding to the pressures of healthcare staff.

### Strengths and limitations

This paper contributes to a growing evidence base supporting efforts to encourage and involve health care staff in research. The findings were strengthened through having research active health care staff involved in reviewing the search terms, screening the papers, extracting the data, and distilling the main benefits. This provided an important ‘sense-check’ throughout the review process. Checking Cohen’s Kappa at key screening stages, a quality check of coding decisions at the data extraction stage, and use of the JBI methodology [39] to guide the process, further helped strengthen the robustness of the review. Nonetheless, we acknowledge that multiple researchers were involved in screening, extraction and coding which introduced a complexity to the process, with potential for slight differences in decision-making (for example, conservative versus less conservative decision-making). Conversely, however, having multiple staff involved also helped tighten the screening and extraction rules through more people reviewing and questioning them. As with any review, we acknowledge the necessary limitations inherent: namely, the records identified are a product of the search strategy and the inclusion and exclusion criteria and the variables selected for extraction. Despite the safeguards reported above, there is potential for relevant records to have been missed from the search strategy or picked up but then excluded during screening. Our focus was relatively narrow—UK context and health care staff. Whilst this was dictated by the context in which the study was undertaken (a collaboration across several NIHR funded Applied Research Collaborations) future research should refine and extend the search to social care staff, covering non-NHS and community settings and/or more conduct more direct, qualitative approach, for example, focussing in on social care recipients of NIHR awards in this area. Indeed, since running the search, which spanned 2004 to February 2023, a paper was published on barriers and enablers to building research capacity in social care, which also found, through interviews, reports of research involvement leading to skills development and enhanced insights into practice, especially from involvement in service improvement projects as part of various post qualification learning programmes [41]. Four additional, more recently published papers have also been identified, reporting on skills development and job satisfaction amongst GPs and other stakeholders [22], on skills development, research career aspirations and improvements to local practice amongst nursing students participating in a locally developed research capacity building initiative [27] and the benefits of networking and building a community within and outside the ambulance service, amongst research paramedics [30]. Finally, we limited the scope of this review by excluding records on disbenefits for staff, whilst barriers and challenges to involvement are reported on in other records, there may be interactions between benefits and disbenefits/barriers that our review will have failed to capture.

## Conclusions

This scoping review provides an evidence base supporting efforts to encourage and involve health care staff in research. It has identified the different ways health care staff are involved in research, the benefits of that involvement, and compared them across different types of involvement. Across 49 records, covering a mix of regions and care settings and professional groups, a consensus group of four research active health care staff distilled six different types of benefits, which include but go beyond career progression. They cover more personal benefits, interpersonal ones which included the opportunity to interact with people from different roles and organisations, and, finally, those influencing their clinical performance and impacting their clinical practice. Records provided examples of people thriving on research, the buffering effects of research compared with the stresses of clinical practice, and the development of communication, project management and leadership skills, alongside research skills, such as literature searching. This in turn raised confidence in their ability to provide evidence-based practice. The insights gained can be used to promote involvement to health care staff, and to highlight to health care organisations the importance of this, in a more comprehensive way, going beyond citing ‘career development’ to also emphasise personal benefits and potential for enhanced job satisfaction. Critically, this applies even where research is not a core component of someone’s job role, as found when comparing the more intense clinical academic forms of involvement with the other less intense forms, with similar patterns found across three of the benefit themes. Heightened job satisfaction and personal fulfilment may contribute to improved staff retention, reducing intense pressure on the UK health care sector with staff shortages and increased demand on services. More research is needed on social care staff involvement in research, and future research should seek to examine more directly the effect on staff wellbeing and retention.

## Supplementary Information

Additional file 1 (Search strategy)

Additional file 2 (Retained records and key characteristics)

## Data Availability

No datasets were generated or analysed during the current study.
