# Supplementary material for: The benefits for health care staff of involvement in applied health research: a scoping review
Source: Health Res Policy Syst. 2025 Aug 18;23:104. doi: 10.1186/s12961-025-01365-1 (PMC12359832; doi:10.1186/s12961-025-01365-1)
Supplement: Supplementary file 1 — Additional file 1 (Search strategy) [file 12961_2025_1365_MOESM1_ESM.doc]

**Additional file 1**

**Search strategy**

**Ovid MEDLINE(R) ALL <1946 to February 08, 2023>**

1 Nurs*.tw.

2 midwi*.tw.

3 (Allied health adj (profession* or personnel or occupation*)).tw.

4 Art therap*.tw.

5 (dental adj (hygienist or nurs* or techn* or therapist)).tw.

6 Dentist*.tw.

7 (dieti*ian* or dietetic).tw.

8 healthcare profession*.tw.

9 health care profession*.tw.

10 ((drama or music) adj therap*).tw.

11 occupational therap*.tw.

12 orthoptist*.tw.

13 (operating adj (department practitioner* or room technician*)).tw.

14 osteopath*.tw.

15 podiatr*.tw.

16 chiropod*.tw.

17 clinician*.tw.

18 prosthetist*.tw.

19 health visitor*.tw.

20 healthcare practitioner*.tw.

21 health care practitioner*.tw.

22 orthotist*.tw.

23 paramedic*.tw.

24 physiotherap*.tw.

25 radiographer*.tw.

26 speech therap*.tw.

27 clinical psycholog*.tw.

28 health* scientist*.tw.

29 pharmacist*.tw.

30 (pharmacy adj (assistant* or technician*)).tw.

31 AHP*.tw.

32 HCP*.tw.

33 care providers*.tw.

34 doctor*.tw.

35 consultan*.tw.

36 GP*.tw.

37 social worker*.tw.

38 impact*.tw.

39 benefit*.tw.

40 (beneficial* or evaluat* or assess*).tw.

41 skill*.tw.

42 development*.tw.

43 advantage*.tw.

44 consequen*.tw.

45 wellbeing*.tw.

46 (capacit* or culture*).tw

47 (clinical adj (academic* or scientist* or investigator*)).tw.

48 fellow*.tw.

49 (research* adj (position* or role* or career* or training or intern* or champion* or advocate*)).tw.

50 ((applied or allied) adj (health research* or healthcare research* or health services research* or social care research*)).tw.

51 mentor*.tw.

52 process evaluation.tw.

53 (exp United Kingdom/ or (national health service* or nhs*).ti,ab,in. or (english not ((published or publication* or translat* or written or language* or speak* or literature or citation*) adj5 english)).ti,ab. or (gb or "g.b." or britain* or (british* not "british columbia") or uk or "u.k." or united kingdom* or (england* not "new england") or northern ireland* or northern irish* or scotland* or scottish* or ((wales or "south wales") not "new south wales") or welsh*).ti,ab,jw,in.) not ((exp africa/ or exp americas/ or exp antarctic regions/ or exp arctic regions/ or exp asia/ or exp oceania/) not (exp united kingdom/ or europe/))

54 1 or 2 or 3 or 4 or 5 or 6 or 7 or 8 or 9 or 10 or 11 or 12 or 13 or 14 or 15 or 16 or 17 or 18 or 19 or 20 or 21 or 22 or 23 or 24 or 25 or 26 or 27 or 28 or 29 or 30 or 31 or 32 or 33 or 34 or 35 or 36 or 37

55 38 or 39 or 40 or 41 or 42 or 43 or 44 or 45 or 46

56 47 or 48 or 49 or 50 or 51 or 52

57 53 and 54 and 55 and 56

58 limit 58 to (english language and humans and yr="2004 -Current")

**Grey literature search**

1. **Search of Google:** ‘the benefits for health and social care staff of involvement in applied health and social care research’; 2004 onwards. Followed by ‘the benefits of involvement for health and social care staff in research’; 2004 onwards.
2. **Google Scholar:** ‘the benefits for health and social care staff of involvement in applied health and social care research’; 2004 onwards:
3. **Targeted websites**
   - **NIHR**
   - **CAHPR**
   - **UKRI**
   - **Council of Deans of Health Clinical Academic Roles Implementation Network (CARIN)**
   - **Department of Health and Social Care**
   - **Health Education England**
   - **The King’s Fund**
   - **THIS Institute**
   - **The Health Foundation**
   - **Florence Nightingale Foundation**
